# From pathogen defence to precision health infrastructure: mRNA vaccines beyond infectious disease in preventive health, chronic disease management, and population wellbeing

**DOI:** 10.3389/fbioe.2026.1869761

**Published:** 2026-08-04

**Authors:** Okechukwu Paul-Chima Ugwu, Chinyere Nneoma Ugwu, Godson Emeka Anyanwu, Mariam Basajja

**Affiliations:** 1 Department of Research, Publication and Extension, Kampala International University, Kampala, Uganda; 2 Department of Anatomy, Faculty of Biomedical Sciences, Kampala International University, Kampala, Uganda

**Keywords:** autoimmune disease, cancer immunotherapy, chronic disease, lipid nanoparticles, mRNA vaccines, pharmacovigilance, precision medicine, preventive health

## Abstract

Messenger RNA (mRNA) vaccine technology is often portrayed as a pandemic-era innovation for infectious disease control, but this perspective no longer reflects its expanding clinical and societal significance. Although global attention intensified during the SARS-CoV-2 pandemic, mRNA platforms are founded on more than three decades of pre-pandemic research in cancer immunotherapy and experimental vaccinology. Emerging evidence demonstrates that programmable RNA technologies can extend beyond pathogen prevention into oncology, immune modulation, protein replacement, cardiovascular risk reduction, and population-level preventive medicine. Nevertheless, existing literature remains fragmented across disciplines and lacks a unifying framework explaining how a single platform can simultaneously support prevention, therapy, and health-system resilience. This narrative review addresses this gap by synthesising literature from PubMed, Scopus, and Web of Science, emphasising studies published between 2018 and 2025 while retaining seminal pre-2018 mechanistic contributions. We integrate mechanistic, translational, and policy evidence to propose that mRNA vaccines should be reconceptualised as adaptive health infrastructure rather than single-purpose biologics. We introduce the Adaptive Preventive Therapeutics Continuum (APTC), a framework positioning mRNA intervention along a spectrum from anticipatory prevention to chronic disease management and precision restoration of physiological function. The central innovation of mRNA technology lies not only in rapid manufacturing but also in its programmable temporality the capacity to generate transient, titratable, and updateable biological responses tailored to disease stage and population need, thereby supporting resilient and adaptable healthcare systems.

## Introduction

The global success of severe acute respiratory syndrome coronavirus 2 (SARS-CoV-2) mRNA vaccines transformed a previously specialised technology into a central pillar of modern biomedicine (Pardi et al., 2018; Chaudhary et al., 2021; Barbier et al., 2022). It is important to recognise, however, that this technology did not emerge solely in response to the pandemic. The conceptual and experimental groundwork was laid over the preceding three decades, beginning with the demonstration that in vitro-transcribed mRNA could direct protein expression *in vivo*, and maturing through sustained programmes in dendritic-cell cancer immunotherapy, personalised neoantigen vaccination, and prophylactic vaccine candidates against influenza, rabies, and other pathogens (Şahin et al., 2014; Pardi et al., 2018). The critical enabling discovery, the use of modified nucleosides to attenuate innate immune sensing, was reported well before SARS-CoV-2 and constituted the scientific foundation on which the pandemic vaccines were built. Framing mRNA technology as a pandemic-born innovation therefore understates both the depth of its scientific lineage and the breadth of its original therapeutic ambitions, which were oncological and protein-replacement oriented long before they were applied to a respiratory pathogen.

Yet public and scientific discourse still commonly treats mRNA vaccines as products designed primarily for infectious disease outbreaks. That assumption now constrains innovation. Multiple pipelines demonstrate that mRNA platforms can encode tumour neoantigens, therapeutic proteins, tolerogenic signals, metabolic regulators, and tissue repair mediators, thereby extending their relevance far beyond classical immunisation (Qin et al., 2022; Rohner et al., 2022; Parhiz et al., 2024). Recent clinical landscape analyses confirm substantial diversification of mRNA development programmes across oncology, rare disease, inflammatory disorders, and cardiometabolic conditions. The unresolved controversy is conceptual rather than technical: are mRNA vaccines simply another vaccine class with broader indications, or do they represent a new category of programmable intervention that dissolves boundaries between prevention and treatment? Existing reviews typically catalogue applications by disease area. Few explain why one platform can plausibly address such heterogeneous conditions, and fewer still examine how this changes preventive health systems, reimbursement logic, and population wellbeing strategies (Huang et al., 2022; Wang et al., 2023; Zhang et al., 2023).

This review advances the central thesis that mRNA technologies are best understood as adaptive health infrastructure capable of generating timed biological instructions across the life course. Their transformative feature is programmable temporality: transient expression that can be rapidly redesigned, repeatedly deployed, and calibrated to evolving risk. This enables a continuum from prophylaxis to disease modification and functional restoration. Critically, the platform’s versatility is inseparable from its engineering substrate: lipid nanoparticle (LNP) delivery systems, ionisable lipid chemistry, nucleoside modification, and tissue-targeting strategies are the enabling technologies that determine whether any given clinical application is feasible. This review integrates delivery science into its synthesis rather than treating it as a peripheral concern.

## Methodology

This review is conducted as a narrative review with a clearly defined purposive sampling strategy. Searches were conducted in PubMed, Scopus, and Web of Science between August and October 2025. Additional hand-searching of references and major journals in vaccinology, immunology, oncology, cardiology, nanomedicine, and health policy was undertaken. The search strategy combined controlled vocabulary and keywords using Boolean logic. Core terms included “mRNA vaccine” OR “RNA vaccine” AND “cancer” OR “chronic disease” OR “autoimmune” OR “cardiovascular” OR “metabolic disease” OR “population health” OR “preventive medicine” OR “health policy” OR “clinical trial” OR “lipid nanoparticle”. Searches were adapted for each database. Inclusion criteria prioritised peer-reviewed articles, phase 1 to phase 3 clinical trials, observational safety studies, translational studies, systematic reviews, and policy analyses published from 2018 to 2025, with emphasis on 2021 to 2025. Seminal earlier mechanistic studies, including the foundational work on modified-nucleoside mRNA and the first descriptions of mRNA as a drug class, were retained when necessary to preserve the scientific lineage of the field. Exclusion criteria included non-English articles without accessible translation, editorials lacking substantive data, and duplicate records.

We acknowledge that the emphasis on literature from 2021 to 2025 introduces a potential temporal concentration effect. Because the SARS-CoV-2 pandemic dramatically accelerated both research output and publication volume in this field, a search prioritising recent years inevitably oversamples pandemic-era and post-pandemic work and may, if read uncritically, create the impression that mRNA technology is a recent arrival. To mitigate this COVID-19-related publication bias, we deliberately retained seminal pre-pandemic mechanistic and translational studies that established the platform, and we foreground these foundational developments in the narrative rather than allowing the recency-weighted corpus to obscure them. Readers should nonetheless interpret the density of recent citations as partly a reflection of publication dynamics rather than a complete picture of the field’s intellectual history.

Study selection followed purposive logic suited to narrative synthesis. Evidence was sampled to capture breadth across disease domains while prioritising methodological rigour, recency, and relevance to the review thesis. Contradictory findings were deliberately retained for comparative analysis. The following table summarises the methodological overview. The review does not claim systematic exhaustiveness and does not report formal inter-rater reliability, quality appraisal scores, or PRISMA-compliant screening statistics, as these are not standard requirements for narrative conceptual reviews. However, in the interest of transparency, it is noted that searches yielded several hundred candidate records per database, with final inclusion guided by the purposive criteria described above and the thematic priorities of the APTC framework.

**TABLE 1:** Methodological overview.

Domain	Approach
Databases	PubMed, scopus, web of science
Timeframe	2018–2025, with priority given to the last 5 years; seminal pre-2018 studies retained
Study types	Trials, cohort studies, translational studies, reviews, policy analyses
Core focus	Applications of mRNA platforms beyond infectious diseases
Selection principle	Relevance, rigour, recency, and conceptual contribution
Analytical method	Thematic critical synthesis (narrative)

Pre-2018 mechanistic studies were retained selectively to preserve the scientific lineage of the platform and to counter recency-weighted publication bias (Table 1).

## Delivery science: the engineering foundation of clinical versatility

The clinical ambitions described throughout this review depend entirely on the capacity to deliver mRNA payloads to the right tissue, at the right dose, with acceptable safety. LNPs are currently the dominant delivery system for clinical mRNA applications, having been validated at scale through SARS-CoV-2 vaccination. However, the native tropism of LNPs formulated with current ionisable lipid compositions is strongly hepatic: following intravenous administration, most particles accumulate in the liver due to ApoE-mediated uptake (Huang et al., 2022; Kowalski et al., 2019). This liver tropism is desirable for protein replacement therapies such as clotting factor delivery but represents a major engineering barrier for the oncology, autoimmune, cardiometabolic, and neuro-inflammatory applications discussed here.

Several strategies are under active investigation to redirect LNP delivery to non-hepatic tissues. Targeting ligands including antibodies, peptides, aptamers, and small molecules can be conjugated to LNP surfaces to engage cell-type-specific receptors (Kowalski et al., 2019; Huang et al., 2022). Lipid composition, PEGylation density, particle size, and ionisation pK_a_ each influence organ selectivity. Intramuscular and subcutaneous administration routes generate localised immune activation and lymph node targeting that differ substantially from intravenous pharmacokinetics. For tumour-directed applications, selective organ targeting (SORT) technology enables systematic redirection of LNP biodistribution by adding a supplemental lipid component. For lymphocyte-targeted tolerogenic vaccines relevant to autoimmunity, spleen-directed LNP formulations have shown preclinical promise (Wang et al., 2025).

Self-amplifying mRNA (saRNA) represents a distinct engineering approach that reduces required dosing mass by incorporating a replicase sequence enabling intracellular amplification of the mRNA payload. This reduces both manufacturing burden and per-dose antigen load, which may be relevant for reactogenicity management in chronic preventive applications (Bhat et al., 2021; Weng et al., 2020). Nucleoside modification, pioneered by Katalin Karikó and Drew Weissman and integral to approved SARS-CoV-2 vaccines, reduces innate immune sensing of exogenous mRNA by substituting N1-methylpseudouridine for uridine, dramatically improving translational efficiency and tolerability (Pardi et al., 2018; Xu et al., 2020).

Cold-chain requirements remain among the most consequential practical constraints on the global deployment of mRNA technologies, and they merit detailed consideration because they bear directly on equity of access. The underlying problem is biophysical. Both the mRNA payload and the LNP carrier are intrinsically labile: single-stranded mRNA is susceptible to hydrolysis and to degradation by ubiquitous ribonucleases, while the LNP is vulnerable to lipid oxidation, particle aggregation, fusion, and loss of encapsulation efficiency. The most degradation-prone event is hydrolytic cleavage of the mRNA backbone, which is accelerated by elevated temperature, trace metal ions, and suboptimal pH, and which directly reduces the proportion of intact, translatable transcript. These chemistries explain why the first-generation SARS-CoV-2 mRNA vaccines required storage at deep-freeze temperatures, a requirement that complicated distribution even in high-income settings and proved especially burdensome where reliable ultra-cold infrastructure is scarce.

Several converging strategies are being pursued to address this. Lipid composition optimisation, including the design of more hydrolytically stable ionisable lipids and antioxidant-protected formulations, aims to slow degradation at the carrier level. Lyophilisation (freeze-drying) seeks to arrest hydrolytic chemistry by removing water and yielding a dry powder that can be reconstituted at the point of care, although the freeze-drying and reconstitution steps themselves can stress the particle and require carefully chosen cryoprotectants and lyoprotectants such as sucrose or trehalose. Thermostable excipient systems, buffer optimisation, and emerging approaches such as spray-drying and encapsulation in more robust matrices are likewise under investigation. The current state of development is best described as promising but pre-mature: several candidate formulations have demonstrated extended stability at refrigerated or even ambient temperatures in preclinical and early studies, yet none has yet displaced the cold chain at scale for licensed products, and regulatory stability data over multi-year horizons remain limited.

The practical implications for low- and middle-income countries (LMICs) are substantial. Ultra-cold storage demands a continuous supply of electricity, specialised freezers, trained personnel, and validated temperature monitoring across long and often fragmented distribution chains, all of which are unevenly available in resource-constrained settings. Where these are lacking, vaccine wastage rises, last-mile delivery to rural and peri-urban populations becomes impractical, and the populations with the greatest disease burden are systematically disadvantaged. For any future chronic preventive mRNA programme, which by definition would require repeated dosing of large, often asymptomatic populations over years, the logistical and economic weight of an unbroken ultra-cold chain would be prohibitive in many LMIC contexts. Progress in thermostabilisation is therefore not a peripheral engineering convenience but a prerequisite for equitable access; without it, the platform risks reproducing the same geographic concentration of benefit that characterised early pandemic-era vaccine distribution.

## The end of the prevention-treatment divide

Modern medicine historically separates prevention from treatment. Vaccines prevent disease in healthy populations, whereas therapeutics treat established pathology (Gu et al., 2022; Zhang et al., 2023). mRNA platforms challenge this binary because the same manufacturing backbone can encode qualitatively different biological instructions (Huang et al., 2022; Qin et al., 2022; Shi et al., 2024). A prophylactic influenza construct, a personalised melanoma neoantigen vaccine, and an autoimmune tolerance candidate differ in payload but share design logic and production workflows (Beck et al., 2021; Liu et al., 2023; Sayour et al., 2024).

This matters because health systems are organised around outdated categories. Preventive budgets are often siloed from specialty care budgets. Reimbursement for one-time vaccination differs from repeated chronic disease management (Gu et al., 2022; Zhang et al., 2023). Regulatory pathways distinguish vaccines from therapeutics even when mechanisms overlap (Li et al., 2023; Lorentzen et al., 2022; Yaremenko et al., 2025). It should be acknowledged that, in the specific context of mRNA-based technologies, the distinction between a vaccine and a therapeutic is itself becoming increasingly blurred. Therapeutic cancer vaccines, tolerogenic constructs, and immune-modulating interventions do not fit neatly into either traditional category: they are administered to individuals with established or incipient disease, yet they act through the antigen-presentation logic historically associated with prophylactic vaccines. While formal regulatory pathways remain distinct, many emerging mRNA platforms occupy a translational space that bridges the conventional preventive and therapeutic categories, and the persistence of rigid classification is increasingly a regulatory and reimbursement artefact rather than a biological reality. As indications diversify, this category rigidity becomes a barrier to efficient translation. The conceptual shift is therefore from product classification to functional classification: the key question should not be whether an intervention is a vaccine or a therapeutic, but whether it reduces future disease burden through programmable biological instruction. Under this lens, cancer recurrence prevention after surgery and autoimmune relapse prevention become forms of advanced prevention (Miao et al., 2021; Haghmorad et al., 2025; Shariati et al., 2024).

## mRNA oncology: the leading proof of concept beyond infection

Cancer provides the strongest evidence that mRNA platforms can move beyond infectious disease. Personalised neoantigen vaccines use tumour sequencing to identify patient-specific mutations, then encode selected targets to amplify T cell responses (Blass and Ott, 2021; Miao et al., 2021). Early studies in melanoma and other solid tumours have reported encouraging immunogenicity and recurrence-risk signals, particularly when combined with checkpoint blockade (Niemi et al., 2022; Zoroddu and Bagella, 2025). The broader clinical pipeline remains substantial and expanding. The strength of oncology evidence lies in biological plausibility and precision targeting (Xie et al., 2023). Unlike shared tumour antigens, neoantigens are less susceptible to central tolerance, increasing immunogenic potential. Manufacturing speed permits individualised treatment windows after surgery or during minimal residual disease states (Trivedi et al., 2024).

The limitations facing precision cancer vaccines are substantial, but they are most usefully understood not as a list of discrete obstacles but as an interconnected system in which biological, computational, clinical, and logistical constraints reinforce one another. At the biological level, tumour heterogeneity generates subclonal antigenic diversity, so that even a well-designed vaccine may select for escape variants and drive immune evasion; this same heterogeneity also undermines the computational layer, because predictive algorithms for antigen and epitope selection must infer immunogenicity from incomplete and noisy sequencing data, and their imperfect accuracy (Kong, 2025; Floudas et al., 2025) means that some encoded targets will be biologically irrelevant or poorly presented. These upstream biological and computational uncertainties propagate into the clinical evidence base: because antigen selection is imperfect and tumour immunogenicity varies widely between cancer types, many trials remain early phase and rely on surrogate immunological endpoints rather than mature overall-survival data, which makes it difficult to distinguish genuinely effective constructs from those that merely induce measurable but clinically inconsequential responses. Finally, the logistical layer compounds the others: the bespoke, per-patient manufacturing that gives neoantigen vaccines their precision also imposes cost and turnaround-time constraints that limit scalability, and these constraints fall hardest on lower-resource health systems, narrowing the populations in which the approach can realistically be evaluated or deployed. Viewed collectively, these factors describe a translation pathway in which weakness at any one level a heterogeneous tumour, an imperfect algorithm, an immature endpoint, or an unscalable manufacturing process can attenuate the benefit generated at the others. Progress in the field will therefore depend less on solving any single limitation in isolation than on coordinated advances across antigen biology, prediction science, trial design, and manufacturing economics.

An intriguing development is evidence that conventional SARS-CoV-2 mRNA vaccination may prime innate and adaptive pathways that synergise with immune checkpoint inhibitors in some cancers, suggesting broader immune system conditioning effects beyond antigen specificity (Grippin et al., 2025; New et al., 2024). This raises a provocative hypothesis: some benefits of mRNA platforms may arise from programmable immunological state change rather than encoded target recognition alone. These findings should be interpreted cautiously, as they emerge from early and indirect data, but they open a research direction warranting rigorous prospective investigation (Table 2).

**TABLE 2:** Comparative evidence synthesis across domains.

Domain	Evidence maturity	Main strength	Principal limitation	Near-term opportunity
Infectious disease^a^	High	Rapid updateability	Variant escape; uptake fatigue	Universal and combination vaccines
Oncology^b^	Moderate	Precision neoantigen targeting	Heterogeneity; cost; immature survival data	Adjuvant recurrence prevention
Autoimmune disease^c^	Early	Antigen-specific tolerance	Disease complexity; autoantigen uncertainty	Steroid-sparing disease control
Cardiometabolic disease^d^	Early	Intermittent biologic recalibration	High safety bar; myocarditis attribution	Population risk reduction
Healthy ageing^e^	Conceptual to early	Systems prevention	Ethical and access concerns; speculative endpoints	Functional longevity programmes

Superscript letters identify supporting references for each domain.

^a^ Pardi et al. (2018), Chaudhary et al. (2021), and Parhiz et al. (2024).

^b^ Liu et al. (2023), Lorentzen et al. (2022), and Sayour et al. (2024).

^c^ Krienke et al. (2021), Song et al. (2024), and Liu et al. (2025).

^d^ Rohner et al. (2022), Dzau and Hodgkinson (2024), and Chia et al. (2024).

^e^ Chen et al. (2025), Doherty et al. (2025), and Hofer et al. (2025).

## Autoimmune disease: from stimulation to tolerance engineering

The most common perception of vaccines is immune activation. Autoimmune disease requires the opposite: selective downregulation of pathogenic responses while preserving host defence (Verbeke et al., 2022; Song et al., 2024). This appears paradoxical, yet mRNA technology is particularly suited to such precision because sequence design, formulation chemistry, and tissue targeting can reshape immune outcomes (Qin et al., 2022; Shi et al., 2024). Preclinical studies have explored tolerogenic mRNA constructs that promote regulatory pathways, antigen-specific tolerance, or anti-inflammatory mediators. The landmark study by Krienke et al. (2021) demonstrated that a non-inflammatory mRNA vaccine encoding myelin autoantigens could suppress experimental autoimmune encephalomyelitis via regulatory T cell expansion. More recently, Liu et al. (2025) showed that LNP-mediated mRNA delivery of PD-L1 to antigen-presenting cells generated durable tolerogenic effects in autoimmune models. Spleen-targeted tolerogenic mRNA-LNP formulations have demonstrated efficacy in experimental asthma (Wang F. et al., 2025), and engineered immune constructs altering antigen-specific tolerance have conferred durable protection in myelin-driven autoimmunity (Ackun-Farmmer et al., 2024).

Recent reviews describe applications including multiple sclerosis, type 1 diabetes, and inflammatory disorders, alongside engineered protein replacement strategies such as IDO1 variants that suppress T cell-mediated pathology (Chen et al., 2024; Imai et al., 2025; Kenison-White et al., 2025). The strength of this domain is specificity: existing immunosuppressants often produce broad infection risk, whereas antigen-directed RNA tolerance could theoretically reduce collateral immunosuppression (Scott et al., 2023; Song et al., 2024). Yet translational hurdles are high. Autoimmune diseases are heterogeneous, relevant autoantigens may be uncertain, and inappropriate innate activation from formulation components could worsen disease. Long-term durability data are sparse (Verbeke et al., 2022; Dhanasekaran et al., 2024). Real-world observational evidence from rheumatoid arthritis populations receiving COVID-19 mRNA vaccines suggests no major flare signal, which is reassuring for platform safety but does not establish therapeutic efficacy (Furer et al., 2021; Fong et al., 2023). The central insight is that the same platform can activate or calm immunity; this is not inconsistency but evidence of programmability, where biological outcome depends on payload, dose, kinetics, route, and delivery context.

## Cardiovascular and metabolic health: the next frontier

Cardiovascular disease remains the leading global cause of mortality, yet vaccine discourse rarely includes cardiometabolic prevention (Dzau and Hodgkinson, 2024; Chia et al., 2024). RNA platforms could target pathways linked to atherosclerosis, dyslipidaemia, thrombosis, inflammation, fibrosis, or regenerative repair (Boada et al., 2021; Peng et al., 2025). Candidate strategies include transient expression of protective proteins, induction of antibodies against pathogenic mediators, or post-infarct tissue healing signals (Kaur and Zangi, 2020; Magadum et al., 2018; Kumar et al., 2024; Adjmi et al., 2025; Khan et al., 2025; Rohner et al., 2022). Current evidence is earlier-stage than oncology, but pipeline mapping already includes cardiac and metabolic indications (Gao et al., 2023; Soroudi et al., 2024). Cell-specific mRNA therapeutics targeting cardiomyocytes and vascular cells represent an active engineering sub-field (Kishore and Magadum, 2024; Wang et al., 2024).

The appeal is strategic: chronic diseases often require lifelong adherence to daily medications, and intermittent programmable biologics could improve adherence and population impact if durable enough. Yet this field faces the sharpest translational scrutiny. Benefit-risk thresholds are higher in prevention for common chronic disease than in lethal malignancy (Landmesser et al., 2020; Hetherington and Totary-Jain, 2022). Safety tolerance is lower because many recipients are stable or asymptomatic. Repeated dosing economics and long-term reactogenicity require careful study (Dzau and Hodgkinson, 2024; Khan et al., 2025). If successful, mRNA tools could shift chronic disease care from reactive maintenance to episodic biological recalibration.

## Healthy ageing and population wellbeing

Population ageing creates multimorbidity, frailty, immunosenescence, and escalating care costs. Conventional disease-by-disease models are poorly suited to this reality (Chen et al., 2025; Hofer et al., 2025). mRNA platforms may enable multi-domain preventive strategies by periodically updating immune competence, reducing latent inflammatory burden, or restoring deficient proteins associated with age-related decline (Chen et al., 2025; Qin et al., 2022). Age-related changes in immune function substantially alter vaccine immunogenicity and reactogenicity, and dosing strategies for older populations must account for inflammaging, reduced T cell diversity, and impaired germinal centre responses (Doherty et al., 2025; Hofer et al., 2025). Seasonal boosters tailored for older adults, vaccines against chronic viral contributors to frailty, and neuroinflammatory modulation are examples of how RNA technologies could support wellbeing rather than only disease treatment.

It is important to state clearly what this section does not claim: there is no established ‘anti-ageing vaccine’ and the relevant applications discussed here remain at conceptual-to-early translational stages. The opportunity is systems prevention reducing vulnerability across interacting pathways not biological rejuvenation. The caution is ethical and distributive. Enhancement narratives can widen inequity if access is restricted to affluent populations. Public health legitimacy requires prioritising burden reduction, functional independence, and equitable ageing rather than cosmetic longevity markets (Chen et al., 2025). Claims in this domain should be understood as identifying research directions rather than established indications.

## Safety, pharmacovigilance, and long-term surveillance

A credible case for mRNA platforms in chronic and preventive applications requires honest engagement with the platform’s safety profile. The benefit-risk calculus for a preventive intervention in asymptomatic populations is substantially more demanding than for a therapeutic in severe disease, and regulators and clinicians will rightly apply higher safety standards to repeated dosing in healthy individuals.

The most prominent platform-level safety signal to emerge from mass SARS-CoV-2 vaccination is myocarditis and pericarditis, predominantly in young males after the second dose of mRNA vaccines. While absolute rates are low estimated at approximately 1 to 10 cases per 100,000 doses in the highest-risk subgroups this signal demands direct attention in the context of proposed cardiovascular applications and repeated dosing regimens (Kang et al., 2025; Lee et al., 2023). The mechanistic basis of vaccine-associated myocarditis has not yet been fully elucidated. Proposed contributors include innate immune activation and, possibly, molecular mimicry, but the precise relative contributions of the lipid nanoparticle, the nucleoside-modified mRNA, the encoded spike antigen, and the interactions among them remain uncertain and are the subject of ongoing investigation. We therefore deliberately avoid asserting that the signal is definitively a platform-level or formulation-level effect; the current evidence does not permit confident attribution to any single component. This distinction is consequential precisely because the present review extrapolates from infectious-disease experience to future cardiovascular and other non-infectious mRNA applications: until the mechanism is resolved, it cannot be assumed either that a new cardiovascular construct would inherit the myocarditis signal observed with SARS-CoV-2 vaccines or that a different payload and formulation would be free of it. The responsible position is to treat the attribution as genuinely open and to make resolving it a research priority (Kiaie et al., 2022; Lee et al., 2023; Shi et al., 2024).

Reactogenicity local and systemic inflammatory responses including pain at the injection site, fever, fatigue, and myalgia is consistently more pronounced with mRNA vaccines than with many traditional vaccines. For single acute-infection prevention, this trade-off is routinely accepted. For a chronic preventive programme requiring annual or semi-annual dosing, reactogenicity tolerance will depend on individual circumstances, indication severity, and whether thermostable formulations or dose adjustments can reduce the innate immune stimulation. Immunogenicity and reactogenicity exist in a mechanistic tension: the innate stimulation that drives stronger adaptive responses also drives systemic symptoms. Optimising this balance is a central goal of next-generation LNP and nucleoside modification engineering (Lee et al., 2023).

Long-term safety uncertainties are inherent at this stage of the technology’s development. Post-market surveillance systems built for infectious disease vaccines will need to be adapted or augmented for applications in which dosing is repeated over years and in populations with pre-existing cardiometabolic, autoimmune, or oncological vulnerabilities. Pharmacovigilance infrastructure must be designed to separate payload-specific adverse events from platform-specific events and from delivery system-specific events. Longitudinal registries, adaptive surveillance designs, and active monitoring frameworks are strategic necessities for the responsible expansion of mRNA applications beyond infectious disease (Shi et al., 2024; Wang et al., 2023; Zhang et al., 2023). Repeated-dosing immunogenicity including the risk of anti-drug antibodies to the LNP components, tolerance induction to the payload antigen in non-tolerogenic applications, or cumulative reactogenicity requires dedicated study in disease-specific phase 2 and phase 3 trials.

## Critical synthesis: why studies conflict and how evidence matures

Apparent contradictions across the literature often stem from comparing unlike interventions under one label. ‘mRNA vaccine’ can refer to prophylactic pathogen immunisation, individualised cancer therapy, protein replacement, or tolerogenic constructs (Barbier et al., 2022; Wang et al., 2023). These differ in dose, schedule, route, target tissue, recipient health state, and acceptable risk thresholds. A second source of conflict is endpoint mismatch. Infectious disease studies often use short-term efficacy outcomes (Kang et al., 2025; Wang et al., 2023). Chronic disease applications require years of follow-up, biomarker validation, and health-economic modelling. Early enthusiasm may overstate readiness when endpoints are immature. Third, platform effects and payload effects are frequently conflated, and delivery system effects are a further distinct source of variability (Kiaie et al., 2022; Lee et al., 2023). The following table synthesises evidence across the major domains covered in this review.

## Proposed conceptual framework: the adaptive preventive therapeutics continuum

We propose the Adaptive Preventive Therapeutics Continuum (APTC) framework. This model repositions mRNA technologies according to function across time rather than by traditional product category. It is differentiated from prior categorisation schemes, which typically sort mRNA applications by disease area or product class, by its functional and temporal logic. The framework’s distinguishing features are: (1) it positions interventions by their stage-matched relationship to disease risk rather than by indication or product type; (2) it explicitly allows bidirectional movement along the continuum as clinical evidence evolves or patient risk status changes; and (3) it treats delivery engineering capability as a cross-cutting enabling condition rather than a separate variable.

Layer one is anticipatory prevention, where interventions are deployed before disease onset in at-risk populations to reduce future incidence, as seen in infectious disease vaccination or risk-targeted cardiometabolic immunoprevention. Operationally, this layer would be triggered by risk stratification using genomic, biomarker, or epidemiological criteria, and would require regulatory pathways analogous to preventive vaccine authorisation with long-term safety follow-up requirements. Layer two is interceptive control, involving interventions during early or minimal disease states to prevent progression or recurrence, such as post-surgical cancer vaccines or early autoimmune modulation. Implementation here would build on existing minimal residual disease monitoring frameworks in oncology or biomarker-triggered early intervention protocols in autoimmunity. Layer three is restorative precision therapy, where interventions deliver transient proteins or repair signals in established disease, including protein replacement, tissue regeneration, or inflammatory resetting. This layer has the clearest regulatory precedent from approved biologics and gene therapy development.

Across all layers operate three enabling properties: rapid redesign, titratable dosing, and modular manufacturing. These features allow flexibility and movement between layers as clinical evidence evolves. The framework’s central logic is stage-matched programmability: the same platform can be applied across prevention, interception, and restoration because it functions as an instruction-based system rather than a static drug class (Barbier et al., 2022; Rohner et al., 2022; Şahin et al., 2014). The APTC framework does not resolve the evidentiary gaps that exist across these domains; rather, it provides a structured conceptual basis for organising research priorities, regulatory strategy, and health system investment decisions.

**FIGURE 1:**
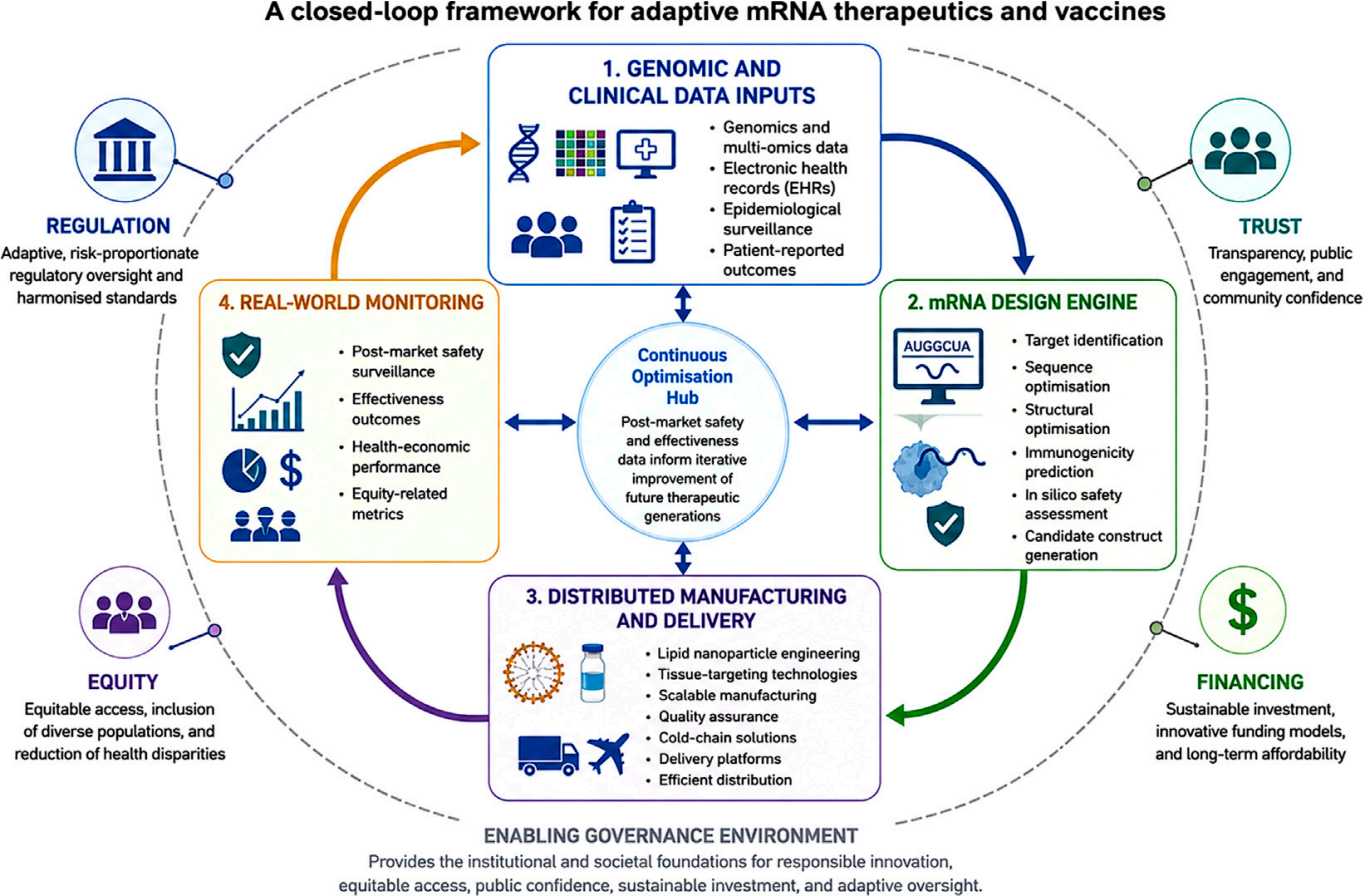
The adaptive preventive therapeutics continuum (APTC framework).

The Adaptive Preventive Therapeutics Continuum (APTC) illustrates a dynamic framework in which preventive and therapeutic interventions are positioned along a spectrum ranging from anticipatory prevention to restorative precision therapy. The anticipatory prevention domain focuses on reducing disease risk before clinical onset and includes examples such as influenza prevention and coronary risk reduction. The interceptive control domain targets early pathological processes to halt, delay, or reverse disease progression, as illustrated by melanoma recurrence control and early rheumatoid arthritis intervention. The restorative precision therapy domain encompasses interventions designed to repair, restore, or optimise biological function in established disease states, including enzyme deficiency correction and post-infarct tissue repair. Indications may move bidirectionally across the continuum as patient risk, disease severity, and therapeutic response evolve over time. Supporting all stages of the continuum are four cross-cutting platform capabilities: redesign speed, scalable manufacturing, dose adjustability, and data-linked updating. These enabling features facilitate rapid therapeutic optimisation, flexible production, personalised dosing strategies, and continuous refinement through integration of real-world clinical data. Collectively, the framework highlights an adaptive approach to preventive therapeutics in which interventions can be continuously modified and repositioned according to changing biological and clinical needs (Figure 1).

**FIGURE 2:**
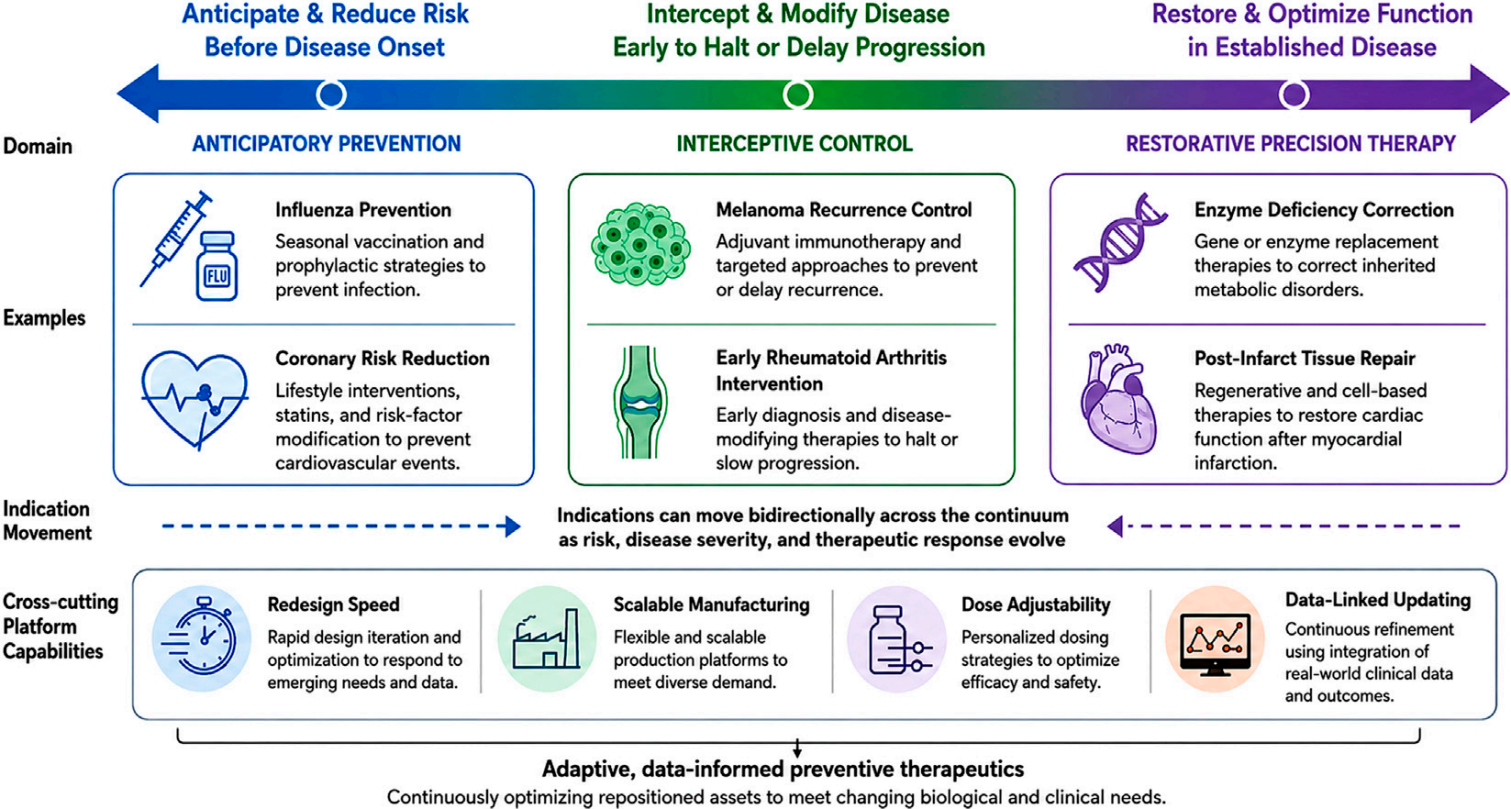
Systems implementation model.

The Systems Implementation Model illustrates a closed-loop framework for the development, deployment, and continuous optimisation of adaptive mRNA therapeutics and vaccines. The model integrates four interconnected operational components arranged in a cyclical workflow. The first component, genomic and clinical data inputs, incorporates genomics and multi-omics datasets, electronic health records, epidemiological surveillance, and patient-reported outcomes to inform therapeutic design. These data are processed through the second component, the mRNA design engine, where target identification, sequence and structural optimisation, immunogenicity prediction, and *in silico* safety assessment are performed to generate candidate therapeutic constructs. The third component, distributed manufacturing and delivery, encompasses lipid nanoparticle engineering, tissue-targeting technologies, cold-chain solutions, and delivery platforms that enable scalable production, quality assurance, and efficient distribution. The fourth component, real-world monitoring, captures post-market safety surveillance, effectiveness outcomes, health-economic performance, and equity-related metrics following deployment. At the centre of the system is a continuous optimisation hub in which post-market safety and effectiveness data are fed back into the design engine to support iterative improvement of future therapeutic generations. Surrounding the operational cycle is an enabling governance environment comprising regulation, equity, trust, and financing. These governance elements provide the institutional and societal foundations necessary for responsible innovation, equitable access, public confidence, sustainable investment, and adaptive regulatory oversight. Together, the model highlights how integrated data systems, platform technologies, manufacturing infrastructure, and real-world evidence can function within a supportive governance framework to enable continuously learning and adaptive mRNA therapeutic ecosystems (Figure 2).

## Discussion

The major implication of this synthesis is that mRNA platforms may become foundational tools for adaptive medicine, but only if development paradigms evolve. Treating each indication as an isolated product underestimates shared infrastructure benefits. Investments in formulation science, cold-chain alternatives, manufacturing nodes, and pharmacovigilance can generate returns across multiple diseases (Barbier et al., 2022; Rohner et al., 2022). Clinically, disease selection should match the strengths of transient expression. mRNA is especially attractive where periodic recalibration is preferable to permanent gene alteration, where rapid updating is valuable, or where multiplex antigen design is advantageous (Chaudhary et al., 2021; Pardi et al., 2018). It may be less suitable when continuous lifelong expression is essential unless repeat dosing becomes highly practical (Parhiz et al., 2024; Qin et al., 2022). The distinction between mRNA platforms and gene therapy platforms is important: mRNA does not integrate into the genome, expression is transient, and the safety profile differs accordingly, though this also means that repeat dosing is required for applications needing sustained effect.

Public trust remains decisive. The success of COVID-19 vaccination demonstrated effectiveness but also exposed polarisation, misinformation, and inequitable access. Expansion into chronic disease will face even greater scrutiny because recipients may perceive lower urgency than during a pandemic, and the safety standard for preventive use in healthy populations is rightly higher. Transparent communication of residual uncertainties including the unresolved mechanistic basis of the myocarditis signal, reactogenicity, and long-term unknowns is not a liability but a prerequisite for durable public trust. Robust long-term surveillance is therefore a strategic necessity, not an optional add-on (Wang et al., 2023; Zhang et al., 2023).

The APTC framework is distinguished from prior classification approaches by its functional and temporal logic rather than categorisation by disease area. However, the framework’s value lies in organising research and policy priorities rather than resolving the substantial evidentiary gaps that remain across most non-infectious applications. Researchers, regulators, and health system planners should engage with this framework as a hypothesis-generating structure that must be tested by rigorous clinical evidence, not as a validated roadmap.

## Implications for clinical practice and policy

For clinicians, mRNA interventions should be considered emerging tools for precision prevention and disease modification rather than only infectious disease immunisation. Multidisciplinary pathways involving oncology, immunology, cardiology, and primary care will be needed (Beck et al., 2021; Liu et al., 2023). Awareness of the platform’s safety profile, including the myocarditis signal and reactogenicity burden, is essential for appropriate patient selection and counselling. For regulators, indication-specific pathways must preserve rigour while recognising common platform components. Platform master files, adaptive manufacturing review, and post-authorisation evidence frameworks could accelerate safe translation (Rohner et al., 2022; Shi et al., 2024). For health systems, procurement models should shift from single-product purchasing to platform capacity investment. Regional manufacturing hubs can strengthen preparedness and routine care simultaneously (Webb et al., 2022). For global policy, equitable licensing, thermostable formulations, and distributed production are essential to prevent a repeat of pandemic-era concentration of supply (Huang et al., 2022; Wang et al., 2023).

## Future research agenda

Future studies should determine optimal durability thresholds for chronic disease indications and identify when repeat dosing is clinically acceptable. Comparative trials should test mRNA against monoclonal antibodies, small molecules, and gene therapies rather than placebo alone (Parhiz et al., 2024; Wang et al., 2023). Precision delivery to non-hepatic tissues remains a major engineering priority requiring systematic work on targeting ligands, SORT technology, saRNA platforms, and tissue-specific ionisable lipid compositions (Huang et al., 2022; Kowalski et al., 2019). Longitudinal registries must separate payload-specific from platform-specific from delivery system-specific adverse events (Shi et al., 2024). Mechanistic investigation of the myocarditis signal and its relationship to LNP composition, dosing schedule, and recipient demographics is urgently needed to inform repeat-dosing safety in non-infectious applications; resolving the relative contributions of the delivery platform, the encoded antigen, and their interaction is a prerequisite for confident extrapolation to cardiovascular indications. Behavioural science research should examine acceptance of preventive RNA interventions outside crisis contexts. Economic evaluations should measure cross-indication returns from shared manufacturing ecosystems (Rohner et al., 2022; Webb et al., 2022). Finally, the APTC framework should be subjected to empirical operationalisation: prospective studies should test whether the stage-matched deployment logic it proposes yields better health outcomes than indication-by-indication development.

## Conclusion

mRNA vaccines have outgrown the conceptual boundaries of vaccinology. Their deepest innovation is not speed alone, but the ability to generate updateable, transient, and stage-specific biological instructions via a programmable delivery platform built on three decades of pre-pandemic scientific development. This allows one platform to operate across prevention, disease interception, and restorative care, but only if the engineering challenges of tissue targeting, cold-chain logistics, and repeated-dosing safety are resolved, and only if the safety requirements appropriate to preventive use in asymptomatic populations are rigorously met. The safety profile of mRNA platforms, including the not-yet-fully-elucidated myocarditis signal, reactogenicity, and long-term unknowns, must be engaged honestly rather than minimised. The APTC framework proposed here offers a conceptual organising structure for research priorities and health system investment decisions, not a validated clinical pathway. If governed wisely and developed with rigorous safety science, mRNA technologies can help redesign healthcare from episodic reaction to adaptive prevention. The future question is no longer whether mRNA can move beyond infectious disease. It is whether institutions can move beyond outdated categories and whether the safety science, delivery engineering, and equity infrastructure can be built quickly enough to realise the platform’s full public health value.
